# The value of enhanced CT features and texture-signatures in assessing the inflammatory infiltration of pancreatic ductal adenocarcinoma

**DOI:** 10.3389/fonc.2023.1078861

**Published:** 2023-02-03

**Authors:** Fangqing Wang, Hang Guo, Shunjia Li, Jianwei Xu, Dexin Yu

**Affiliations:** ^1^ Department of Radiology, Qilu Hospital of Shandong University, Jinan, China; ^2^ Department of Radiology, Laiyang Central Hospital of Yantai, Yantai, China; ^3^ Department of Radiation Oncology, Qilu Hospital of Shandong University, Jinan, China; ^4^ Department of Pancreatic Surgery, Qilu Hospital of Shandong University, Jinan, China

**Keywords:** pancreatic ductal adenocarcinoma, inflammatory infiltration, computed tomography features, computed tomography texture features, predictive value

## Abstract

**Purpose:**

To explore the predictive value of computed tomography (CT) imaging features and CT-based texture analysis in assessing inflammatory infiltration in pancreatic ductal adenocarcinoma (PDAC).

**Methods:**

A total of 43 patients with PDAC confirmed by surgical pathology were included in the study. The clinical, radiological, surgical, and pathological features of the patients were analyzed retrospectively using the chi-square test or Spearman’s correlation. Receiver operating characteristic (ROC) curves were utilized to assess the overall predictive ability of the tumor enhancement degree on triphasic contrast-enhanced CT images for the inflammatory infiltration degree in PDAC. Furthermore, all CT data were uploaded to the RadCloud platform for region of interest (ROI) delineation and feature extraction. Then, the Variance Threshold and SelectKBest algorithms were used to find the optimal CT features. Binary logistic regression was employed to analyze the selected features in all three contrast-enhanced CT phases, and regression equations were formulated. ROC analysis was performed to evaluate the predictive effectiveness of each equation.

**Results:**

The analysis revealed a statistically significant correlation between the degree of differentiation and radiological findings such as necrosis and cystic degeneration, vascular invasion, and the presence of ascites (*P* < 0.05). The enhancement degree of the tumor in both the arterial and venous phases was significantly correlated with the inflammatory infiltration degree (*P* < 0.05); however, the areas under the ROC curve (AUCs) of arterial and venous enhancement were 0.570 and 0.542, respectively. Regression equations based on the texture features of triphasic contrast-enhanced tumors were formulated, and their AUCs were 0.982, 0.643, and 0.849.

**Conclusion:**

Conventional radiological features are not significantly correlated with the degree of inflammatory infiltration in PDAC. The enhancement degrees in both the arterial phase and venous phase were statistically correlated with the inflammatory infiltration level but had poor predictive value. The texture features of PDAC on contrast-enhanced CT may show a better assessment value, especially in the arterial phase.

## Introduction

Pancreatic ductal adenocarcinoma (PDAC) has a poor prognosis, with a 5-year survival rate of less than 5% (1, 2) and an 80% recurrence rate after surgery (1); therefore, is considered an extremely lethal disease. The microenvironment of PDAC is closely related to its dismal prognosis, and inflammation is an essential aspect of the tumor (3). It has been demonstrated that inflammation plays a key role not only in the development of PDAC but also in determining patient prognosis (4). Therefore, understanding the inflammatory activities within the microenvironment of primary PDAC can provide meaningful information for its diagnosis, treatment, and prognosis (5). The current gold standard for the detection of inflammatory activity in PDAC remains histological evaluation with biopsy. However, this procedure has some drawbacks such as the invasiveness of the sampling, the difficulty in performing the puncture, the small size of the extracted specimen, and its contraindications, all of which compromise its clinical application to some extent.

In contrast, imaging modalities such as computed tomography (CT), magnetic resonance imaging (MRI) and positron emission tomography/computed tomography (PET/CT) are noninvasive and convenient, and the inflammatory state within PDAC can be evaluated by measuring certain imaging features. For instance, correlations between pancreatic inflammatory lesions and the degree of delayed intensification were reported by Ren and Axsom (6, 7). Especially in recent years, a massive number of quantitative features can be extracted from radiological images with a variety of algorithms, and radiomics and texture analysis are expected to be feasible techniques for assessing inflammatory infiltration within PDAC (8, 9). However, to the best of our knowledge, no study has been conducted on imaging evaluation of inflammatory infiltration in PDAC. In this study, we investigated the value of traditional CT imaging by extracting texture features for the assessment of inflammatory infiltration in PDAC with the aim of providing more accurate information for the precise diagnosis and treatment of this disease.

## Materials and methods

### Patient data

**Figure 1:**
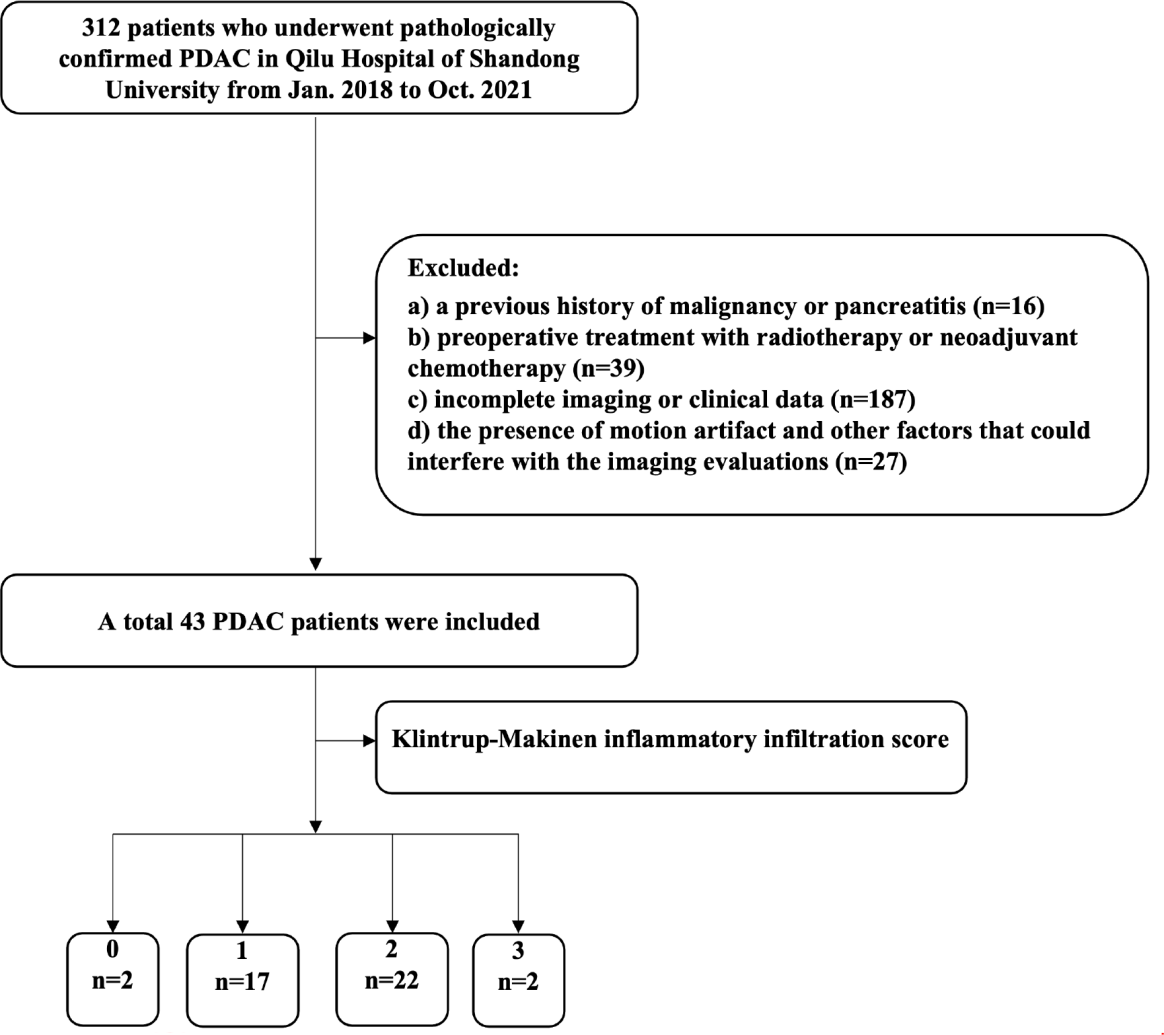
Flow chart visualizing the patient selection process.

Institutional review board approval was obtained for this retrospective study, and the requirement for written informed consent was waived. Patients with pathologically proven PDAC who underwent CT scanning between January 2018 and October 2021 at Qilu Hospital of Shandong University were reviewed. The patient inclusion criteria were: a) contrast-enhanced CT was performed within 2 week before surgery, with complete clinical data; b) all tumors surgically resected and confirmed as PDAC by postoperative pathology. The exclusion criteria were as follows: a) a previous history of malignancy or pancreatitis; b) preoperative treatment with radiotherapy or neoadjuvant chemotherapy; c) incomplete imaging or clinical data; and d) the presence of motion artifact and other factors that could interfere with the imaging evaluations. Thus, 43 patients (31 men and 12 women) were enrolled in this study, with a mean age of 61.2 ± 9.68 years (age range, 34-77). The case screening criteria are shown in **Figure 1**.

### CT imaging

All patients in our study underwent plain and triphasic contrast-enhanced CT examinations with a Philips Brilliance 256-slice spiral CT scanner (Philips Medical Systems, Amsterdam, The Netherlands), with the following scanning parameters: tube voltage of 120 kV, tube current of 200 mAs, slice thickness of 5.0 mm, and slice interval of 5.0 mm. For contrast-enhanced CT examination, all patients were injected with the contrast agent iopromide at a concentration of 300 mg/mL (Ultravist, Bayer Healthcare, Berlin, Germany) and a flow rate of 3.0 mL/s *via* the cubital vein. Arterial, venous, and delayed phase scans were performed at 30 s, 60 s, and 2-3 min after contrast injection, respectively. The scan scope was from the dome of diaphragm to the lower pole of both kidneys. All patients were scanned in the supine position at maximal inspiration, with arms raised over their heads.

### CT image analysis

The CT images were reviewed by two associate chief radiologists (F.W. and W.J., with 7 and 13 years of experience, respectively) who were blinded to the detailed histopathological information of all patients and the study aim, in consultation with a third chief radiologist (D.Y., with 21 years of experience) in case of disagreement. Qualitative and quantitative analysis included extraction and assessment of the following features: a) tumor size (maximal diameter), b) tumor location (head and neck vs body and tail), c) tumor shape (round vs irregular), d) cystic necrosis, e) vascular invasion, f) peripancreatic fat invasion, g) main pancreatic duct dilatation, h) lymph node enlargement, and i) ascites. The regions of interest (ROIs) were placed in the solid part of the tumor showing the most remarkable enhancement, avoiding areas of the necrosis and calcification and blood vessels were avoided. The CT value was measured three times by placing the largest ROIs of each lesion, and then averaged. The tumor-to-pancreas enhancement ratio was calculated by dividing the CT value (HU) of the pancreatic tumor by that of the pancreatic parenchyma at each contrast-enhanced CT phase.

### CT image texture analysis

**Figure 2:**
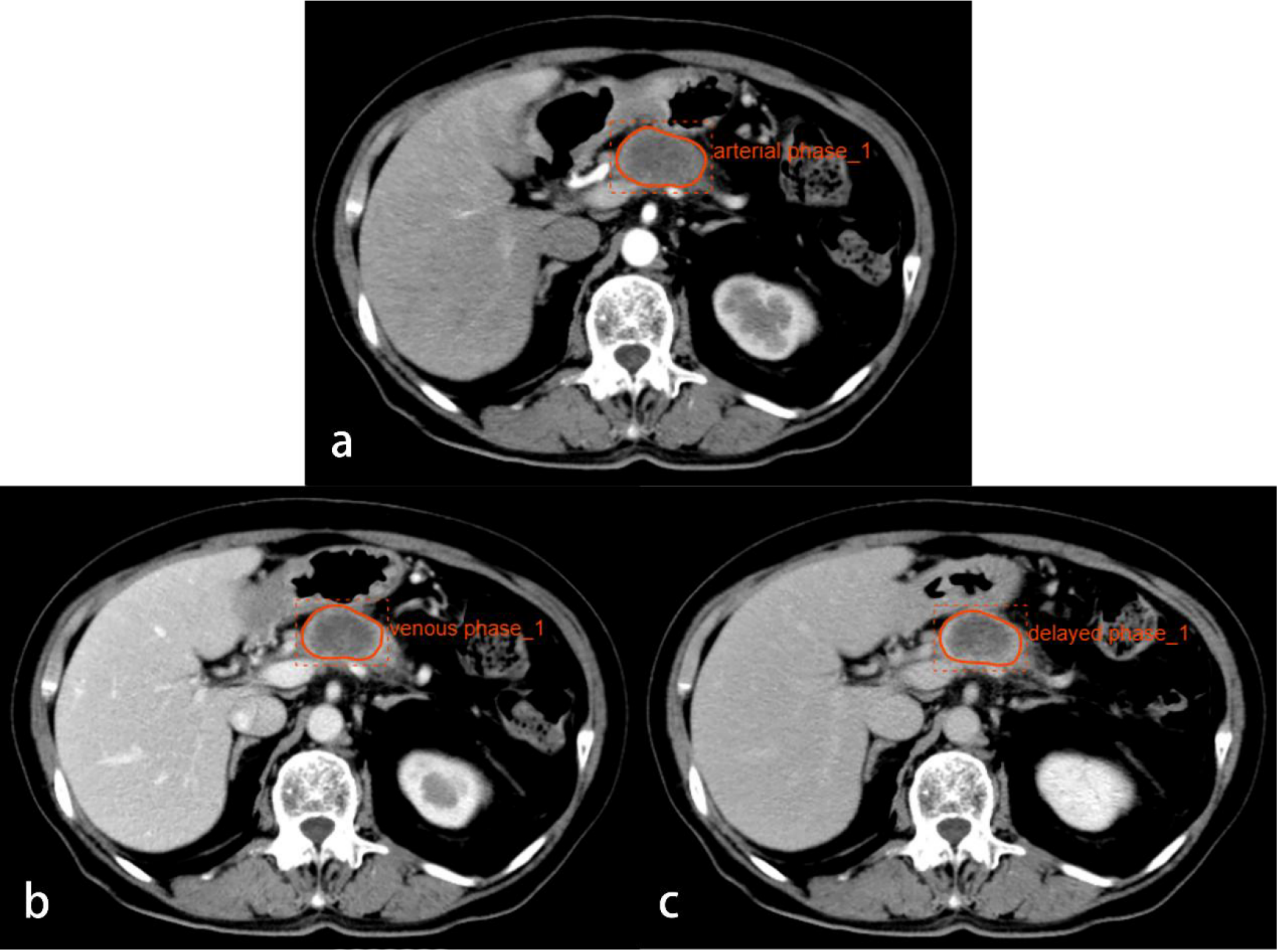
CT images from a 74-year-old woman with PDAC in the neck of the pancreas. Regions of interest are outlined in orange on arterial **(A)**, venous **(B)**, and delayed phase **(C)** images.

The preoperative CT images were exported in DICOM format and uploaded to the RadCloud platform (Huiying Medical Technology Co., Ltd., Beijing, China) for the extraction of texture features. The maximum cross-sections of the tumor in the arterial, venous, and delayed phases were outlined as the ROIs (**Figure 2**). Taking the degree of inflammatory infiltration as the label, the Variance Threshold and the SelectKBest algorithms were used to obtain the optimal features. Logistic regression analysis was performed based on the optimal features from the triphasic enhanced images, and regression equations were established to evaluate the predictive performance of texture features in each phase for inflammatory infiltration.

### Pathological analysis

**Figure 3:**
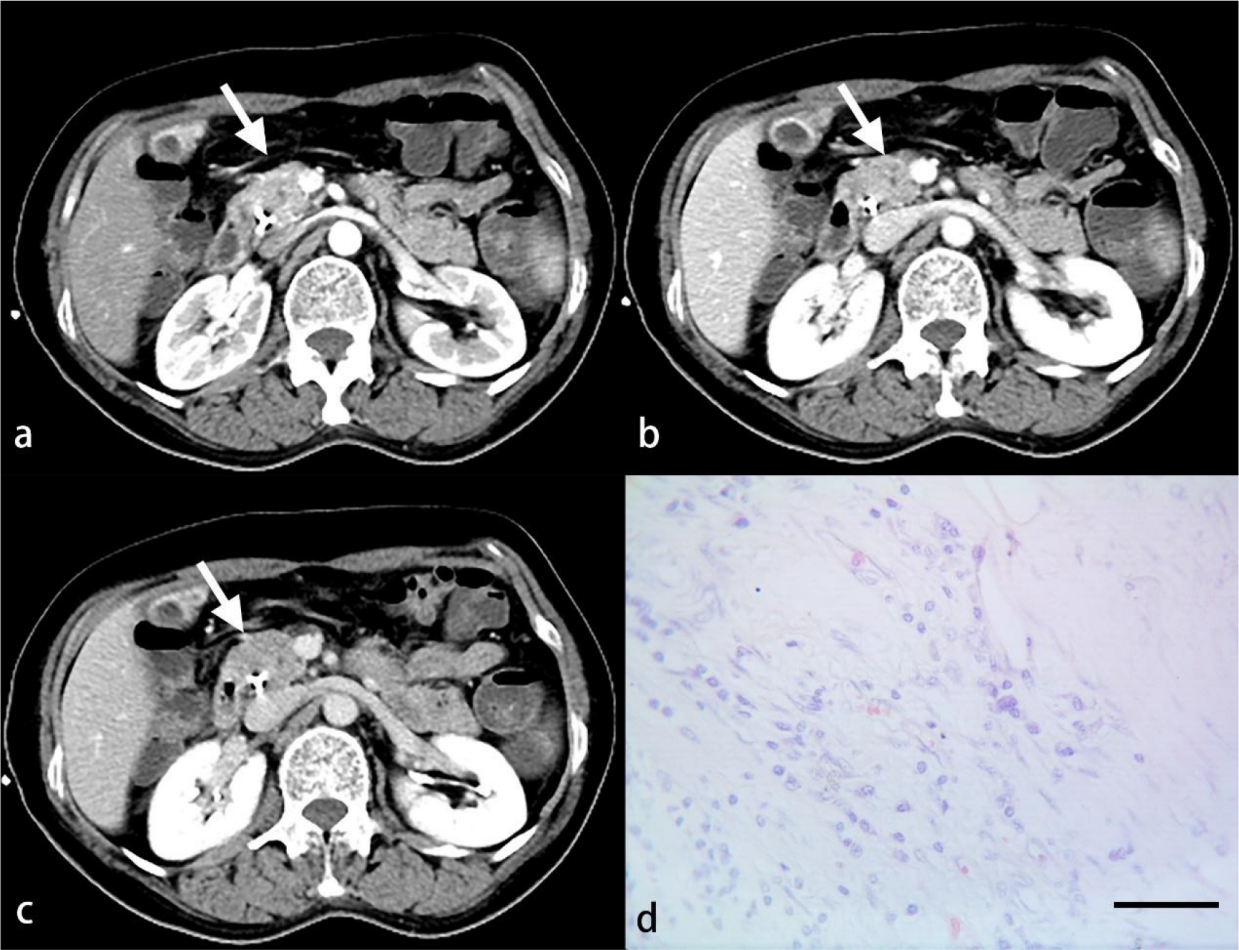
A 52-year-old woman with PDAC. Arterial **(A)**, venous **(B)** and delayed **(C)** phase CT images show an ill-defined mass in the head of the pancreas (arrow). No inflammatory cells are observed under light microscopy **(D)**. (HE, scale bar=50 μm).

**Figure 4:**
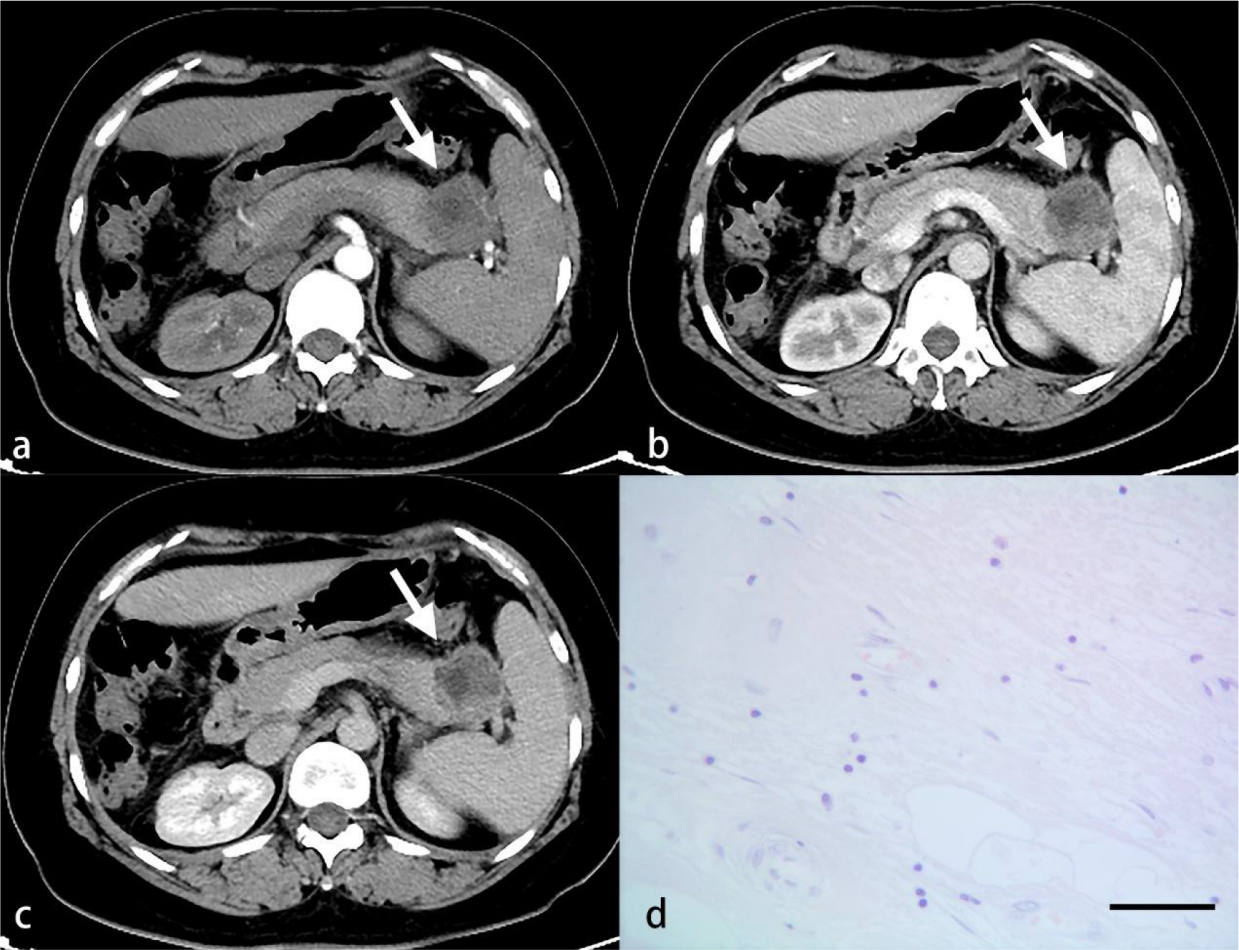
A 56-year-old woman with PDAC. Arterial **(A)**, venous **(B)** and delayed **(C)** phase CT images show a well-defined mass in the tail of the pancreas (arrow). A small number of inflammatory cells are observed under light microscopy **(D)**. (HE, scale bar=50 μm).

**Figure 5:**
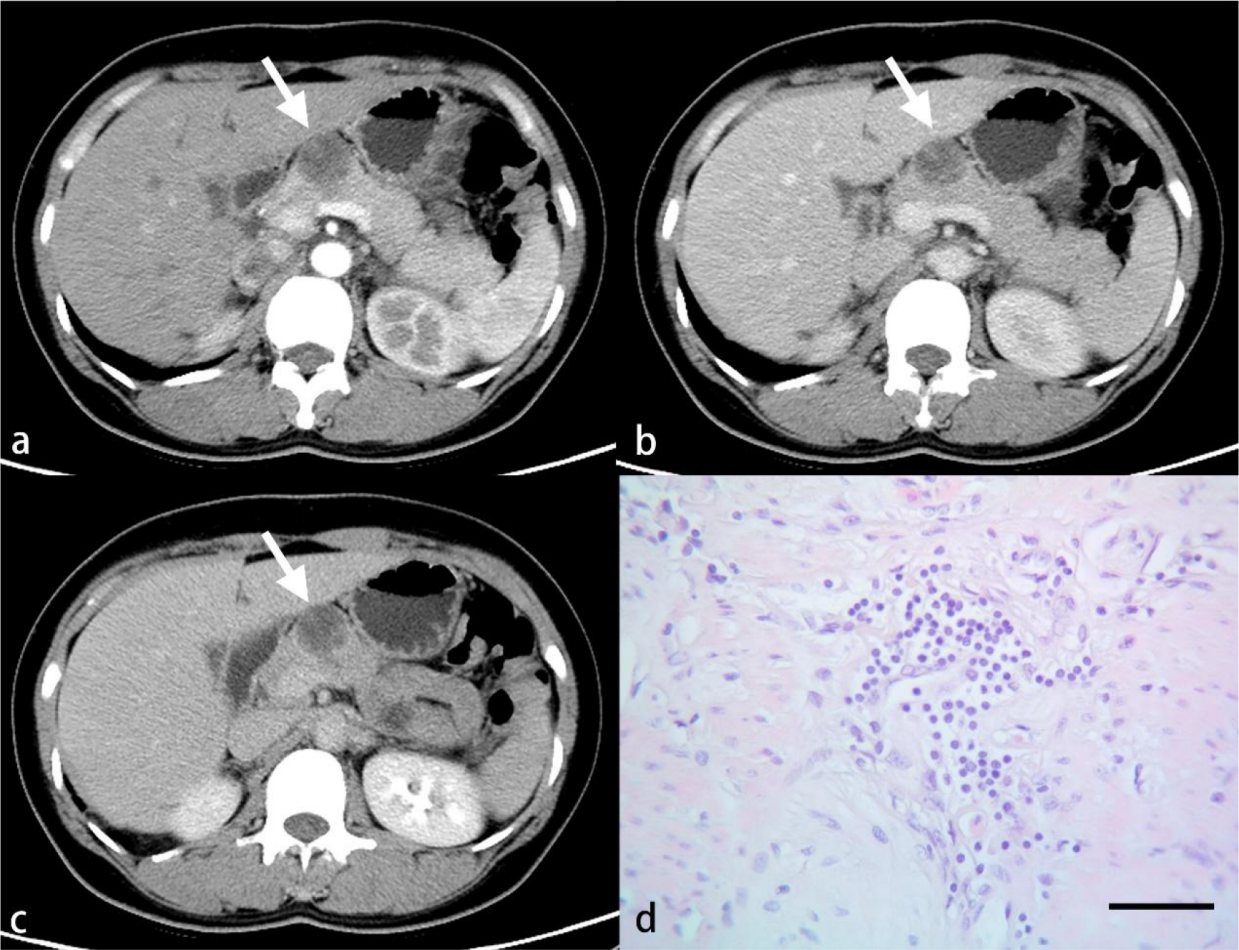
A 46-year-old woman with PDAC. Arterial **(A)**, venous **(B)** and delayed **(C)** phase CT images show a well-defined mass in the neck of the pancreas (arrow). A larger number of inflammatory cells that formed a band-like infiltrate are observed under light microscopy **(D)**. (HE, scale bar=50 μm).

**Figure 6:**
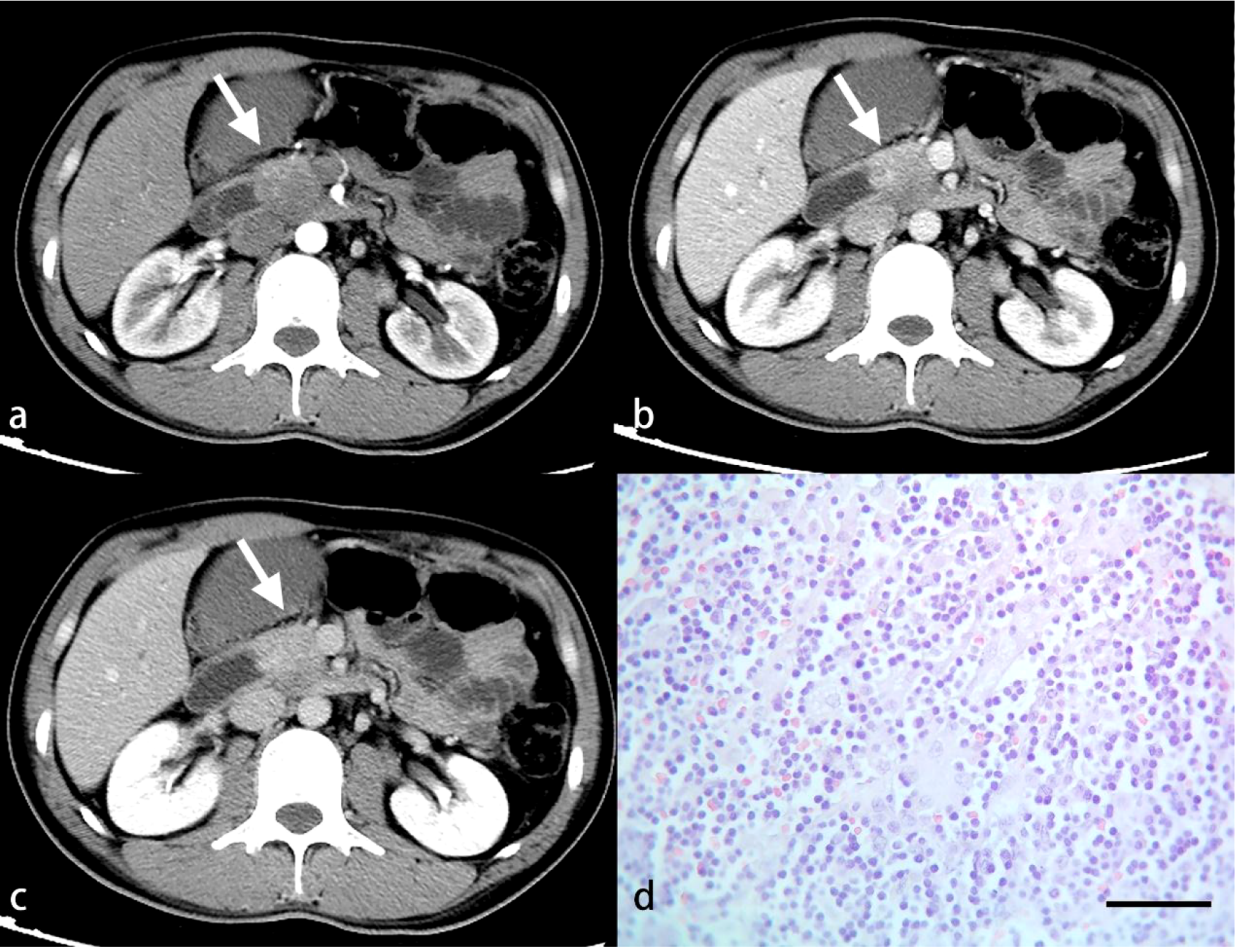
A 34-year-old man with PDAC. Arterial **(A)**, venous **(B)** and delayed **(C)** phase CT images show an ill-defined mass in the neck of the pancreas (arrow). A great number of inflammatory cells are observed under light microscopy **(D)**. (HE, scale bar=50 μm).

Routine HE staining was performed on 43 pancreatic tumor sections, which were divided into three groups according to the degree of differentiation: high, medium, and low. Three random high‐magnification visual fields were randomly selected to comprehensively assess the degree of inflammatory cell infiltration. A four-degree scale based on the study by Klintrup et al. (10) was used to estimate inflammatory cell infiltration: no increase of inflammatory cells was scored as 0 (**Figure 3**), a mild and patchy increase of inflammatory cells was scored as 1 (**Figure 4**), a larger number of inflammatory cells forming a band-like infiltrate was scored as 2 (**Figure 5**), and a significant inflammatory response with a large number of inflammatory cells filling the visual field was scored as 3 (**Figure 6**). To improve the efficiency of the classification system and for ease of analysis, the original four-point scale was reduced to a two-point scale: a score of 0-1 was assigned to the mild inflammatory infiltrate group and 2-3 to the severe inflammatory infiltrate group.

### Statistical analysis

All statistical analyses were conducted using the SPSS software package (version 26.0; IBM Corp., Armonk, NY, USA). The statistical approaches were chosen based on the different data types in this study: we made statistical comparisons using the chi-squared test for categorical variables and Spearman’s correlation for continuous variables. Binary logistic regression was used to analyze the optimal texture features in each phase. Then receiver operating characteristic (ROC) curves were plotted, from which the area under the curve (AUC), sensitivity and specificity were calculated. P values less than 0.05 (*P* < 0.05) were considered statistically significant.

## Results

### Radiologic features

The clinical and radiologic characteristics of a total of 43 PDACs are presented in **Table 1**, with a mean diameter of 3.96 cm ± 1.62 (range, 1.2 – 9.0 cm). Twenty-nine tumors were located in the head and neck of the pancreas, and the other 14 were located in the body and tail. Seventeen lesions were round in shape, and the other 26 were irregular. CT showed pancreatic duct dilatation in 20 lesions, cystic necrosis in 15, adjacent vascular invasion in 20, peripancreatic fat invasion in 20, lymph node enlargement in 15, and ascites in 2. The mean tumor-to-pancreas enhancement ratios of the PDAC tumors in the arterial, venous, and delayed phases were 0.59 ± 0.17, 0.76 ± 0.19, and 0.90 ± 0.18, respectively.

**Table 1:** Correlations between radiologic features and pathological differentiation degrees.

Variables	High Differentiation	Medium Differentiation	Low Differentiation	P Value
Tumor location				0.6†
Head and neck of the pancreas	2	12	15	
Body and tail of the pancreas	2	4	8	
Tumor size				0.404‡
≤4 cm	3	10	13	
>4 cm	1	6	10	
Tumor shape				0.478†
Round	1	5	11	
Irregular	1	11	12	
Cystic necrosis				0.03†
Positive	0	3	12	
Negative	4	13	11	
Vascular invasion				0.031†
Positive	1	4	15	
Negative	3	12	8	
Peripancreatic fat invasion				0.959†
Positive	2	7	11	
Negative	2	9	12	
Pancreatic duct dilatation				0.061†
Positive	2	11	7	
Negative	2	5	16	
Lymph node enlargement;				0.419†
Positive	2	7	6	
Negative	2	9	17	
Ascites				0.000†
Positive	2	0	0	
Negative	2	16	23	

†P values were calculated by the chi-square test.

‡P values were calculated by Spearman’s correlation analysis.

### Pathological features

Generally, PDAC tumors were mostly greyish white or greyish yellow masses with a hard texture. Regarding the differentiation degree, a total of 4, 16, and 23 tumors were identified as demonstrating high, medium, and low differentiation, respectively. Regarding the inflammatory infiltration degree, the Klintrup-Makinen score was 0 in 2 tumors, 1 in 17, 2 in 22, and 3 in 2; 19 and 24 tumors were in the mild and severe inflammatory infiltrate groups, respectively.

### Correlation between radiologic features and pathological features

There were statistically significant correlations between cystic necrosis (*P* = 0.03), vascular invasion (*P* = 0.031), ascites (*P* < 0.001), and the degree of PDAC differentiation. No significant correlation was found for the other radiologic features (*P* > 0.05) (**Table 1**). In addition, there were no significant correlations between any of the aforementioned radiologic features and the degree of inflammatory cell infiltration (**Table 2**).

**Table 2:** Correlations between radiologic features and inflammatory cell infiltration degrees.

Variables	Inflammatory Infiltration Score				P Value
	0	1	2	3	
Tumor location					0.439†
Head and neck of the pancreas	2	12	13	2	
Body and tail of the pancreas	0	5	9	0	
Tumor size					0.922‡
≤4 cm	2	10	12	2	
>4 cm	0	7	10	0	
Tumor shape					0.769†
Round	1	8	7	1	
Irregular	1	9	15	1	
Cystic necrosis					0.327†
Positive	0	5	10	0	
Negative	2	12	12	2	
Vascular invasion					0.21†
Positive	0	10	10	0	
Negative	2	7	12	2	
Peripancreatic fat invasion					0.154†
Positive	1	10	7	2	
Negative	1	7	15	0	
Pancreatic duct dilatation					0.459†
Positive	1	8	9	2	
Negative	1	9	13	0	
Lymph node enlargement;					0.73†
Positive	0	6	8	1	
Negative	2	11	14	1	
Ascites					0.572†
Positive	0	0	2	0	
Negative	2	17	20	2	

†P values were calculated by the chi-square test.

‡P values were calculated by Spearman’s correlation analysis.

### Correlation between PDAC tumor enhancement and pathological features

**Figure 7:**
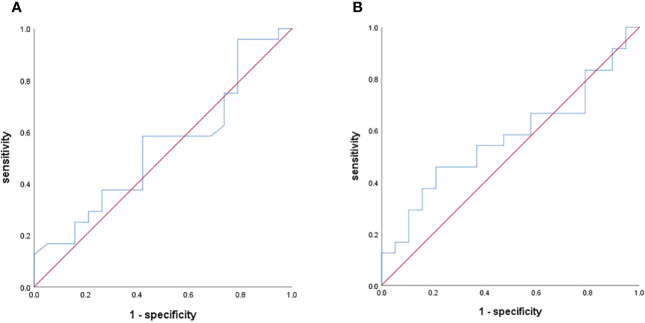
ROC curves for assessing the diagnostic performance of the Tumor-to-pancreas enhancement ratio in the arterial phase **(A)** and venous phase **(B)**.

Spearman’s correlation analysis showed no statistically significant correlations between the tumor-to-pancreas enhancement ratio in the arterial, venous and delayed phases and the differentiation degrees (*P* = 0.823, *P* = 0.951, and *P* = 0.543, respectively). The correlations between the tumor-to-pancreas enhancement ratio in the arterial and venous phases and the inflammatory infiltration score were statistically significant (*P* = 0.017 and *P* = 0.013, respectively), while that between the tumor-to-pancreas enhancement ratio in the delayed phase and the inflammatory infiltration score was not (*P* = 0.688). Then, ROC curves were plotted to assess the predictive value of the tumor-to-pancreas enhancement ratio in the arterial and venous phases on the inflammatory infiltration degree, and the resulting AUCs were 0.542 and 0.570, respectively (**Figure 7**).

### Texture analysis of PDAC tumors

A total of 1409 texture features were extracted from the contrast-enhanced CT images of 43 lesions. Eventually, twelve, six, and eight optimal features were selected by the algorithm for the arterial, venous, and delayed phases, respectively (**Tables 3**–**5**). These optimal features were included in the regression equations after binary logistic regression analysis.

**Table 3:** Optimal texture features from the arterial phase.

No.	Texture Feature	Class	Filter	P Value
X1	Kurtosis	firstorder	wavelet-HLL	0.0011
X2	SizeZoneNonUniformity	glszm	wavelet-LHL	0.0159
X3	LargeDependenceHighGrayLevelEmphasis	gldm	wavelet-LLH	0.0256
X4	Kurtosis	firstorder	wavelet-LLL	0.0267
X5	LargeAreaHighGrayLevelEmphasis	glszm	wavelet-LLH	0.0274
X6	HighGrayLevelZoneEmphasis	glszm	wavelet-HHL	0.03
X7	SmallAreaHighGrayLevelEmphasis	glszm	wavelet-LHL	0.0342
X8	Kurtosis	firstorder	original	0.0421
X9	Kurtosis	firstorder	squareroot	0.425
X10	Kurtosis	firstorder	square	0.0454
X11	Kurtosis	firstorder	exponential	0.0457

**Table 4:** Optimal texture features from the venous phase.

No.	Texture Feature	Class	Filter	P Value
X1	Skewness	firstorder	wavelet-LLL	0.0235
X2	Skewness	firstorder	logarithm	0.0424
X3	Skewness	firstorder	squareroot	0.0426
X4	Skewness	firstorder	original	0.044
X5	Skewness	firstorder	exponential	0.0461
X6	Skewness	firstorder	square	0.0472

**Table 5:** Optimal texture features from the delayed phase.

No.	Texture Feature	Class	Filter	P Value
X1	SmallAreaHighGrayLevelEmphasis	glszm	wavelet-HHL	0.0087
X2	LargeDependenceLowGrayLevelEmphasis	gldm	wavelet-LLH	0.0169
X3	HighGrayLevelZoneEmphasis	glszm	wavelet-LLH	0.0263
X4	LongRunLowGrayLevelEmphasis	glrlm	wavelet-HLL	0.0268
X5	ZoneEntropy	glszm	wavelet-LHH	0.0286
X6	LargeDependenceHighGrayLevelEmphasis	gldm	wavelet-LLH	0.032
X7	RunVariance	glrlm	wavelet-HLL	0.0371
X8	LargeDependenceLowGrayLevelEmphasis	gldm	wavelet-HLL	0.0495

The following equations were constructed based on the optimal texture features from the arterial phase, venous phase, and delayed phase, respectively:


$$\begin{array}{l} \text{Logit}\left(p\right)=30.902-3.707\times \text{X}1+0.191\times \text{X}2+0.012\times \text{X}3+1.401\times \text{X}4-5.75\times X5-2.21\times \text{X}6\\ -207.175\times \text{X}7+787.901\times \text{X}8-793.509\times \text{X}9-57.502\times \text{X}10+267.685\times \text{X}11;\\ \text{Logit}\left(p\right)=0.449+0.657\times \text{X}1+15.843\times \text{X}2-45.751\times \text{X}3+49.931\times \text{X}4-13.604\times \text{X}5-6.242\times \text{X}6; \end{array}$$

Logit(p)=−5.966+2.2×X1−0.863×X2+0.064×X3−1.086×X4+0.034×X5+0.003×X6−0.823×X7+0.313×X8

**Figure 8:**
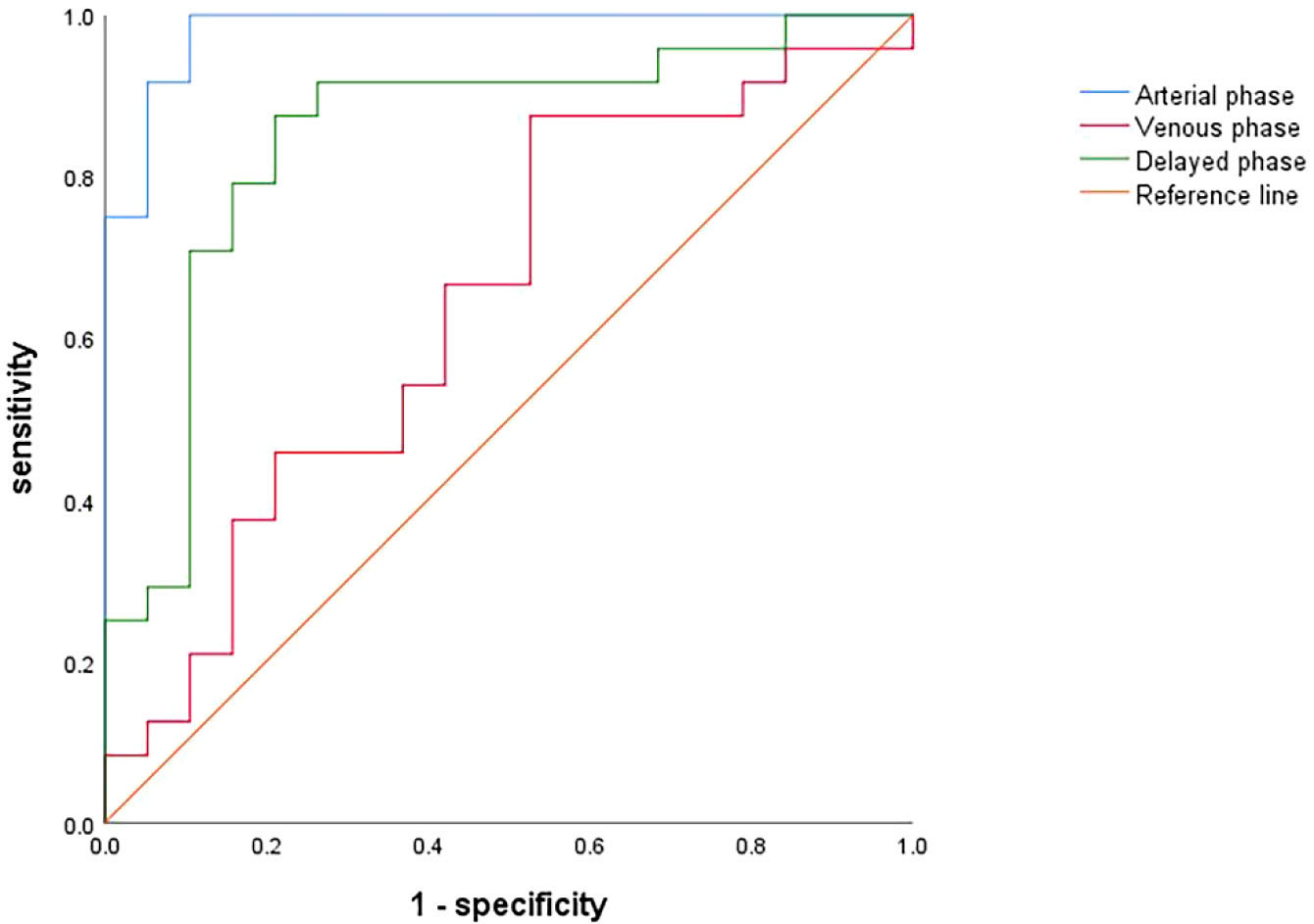
ROC curves for assessing the diagnostic performance of regression equations constructed based on optimal texture features from the arterial (blue), venous (red) and delayed (green) phases.

The predictive performance of texture features from the arterial, venous, and delayed phases was evaluated using the ROC curves of the regression equations: the AUC of the regression equation from the arterial phase features was 0.982, with a sensitivity of 100% and a specificity of 89.5%; the AUC of the regression equation from the venous phase features was 0.643, with a sensitivity of 87.5% and a specificity of 47.4%; and the AUC of the regression equation from the delayed phase features was 0.849, with a sensitivity of 87.5% and a specificity of 78.9% (**Figure 8**).

## Discussion

In recent years, as an inexpensive non-invasive imaging technology, CT has become the mainstay for the diagnosis and evaluation of PDAC (11). The tumor microenvironment has a profound influence on the metabolism and biological behaviors of the tumor itself (12), especially for PDAC, and the current diagnosis and treatment challenges of PDAC are closely related to its particular microenvironment (13). With the rapid development of CT technology, especially when combined with quantitative imaging techniques such as texture analysis, the assessment of the tumor microenvironment using non-invasive imaging technology has become possible (14).

In 1863, Virchow first discovered the presence of immune cell infiltration in neoplastic tissues (15), and it is now generally accepted that chronic aseptic inflammation of the pancreas is an important risk factor for the development of PDAC, along with its initiation, progression, and metastasis (16). As an essential part of the tumor microenvironment, chronic aseptic inflammation disturbs the microenvironment and metabolism of normal pancreatic tissue by accelerating intracellular DNA damage, altering the activity of tumor-related signaling pathways, and inducing the production of inflammatory factors, thus creating favorable conditions for the malignant transformation of normal cells and the proliferation of tumor cells (3). Incio et al. demonstrated that chronic pancreatic inflammation and fibrous tissue proliferation triggered by obesity not only accelerated the progression of PDAC, but was also associated with chemotherapy resistance (17); Aziz et al. measured inflammation-related indicators such as C-reactive protein, the neutrophil-to-lymphocyte ratio, and the platelet-to-lymphocyte ratio in PDAC patients and found that the systemic-immune-inflammation index was an independent predictor of prognosis in PDAC patients (18). Furthermore, Pu et al. found that baicalein, which has anti-inflammatory effects, can slow down or even reverse inflammation-induced pancreatic acinar-to-ductal metaplasia, offering a new idea in effective treatment of PDAC in the clinic (19).

Thus, understanding inflammation in PDAC can provide important information for the diagnosis and evaluation of this lethal disease. However, at present, the assessment of inflammatory activities in tumors is mainly dependent on pathology (20). As the gold standard, pathological assessment has high accuracy, but is also traumatic, expensive, and, in certain circumstances, contraindicated. In clinical practice, on the other hand, the imaging modalities such as contrast-enhanced CT are simple, non-invasive, and repeatable. Therefore, in this study, we used pathological findings as a reference standard, to evaluate the value of contrast-enhanced CT in assessment of inflammatory infiltration in PDAC.

The present study revealed statistically significant correlations between cystic necrosis, vascular invasion, ascites and the pathological differentiation degrees in PDAC, matching the observations by Lee, Mierke, Ariake et al. (21–23); however, correlations between the aforementioned imaging features and inflammatory infiltration were not significant, indicating that conventional imaging might not be sufficient to assess the differentiation degree of PDAC. Regarding the relationship between PDAC tumor enhancement and the degree of inflammatory infiltration, this study demonstrated significant inverse correlations in the arterial and venous phases. The inverse correlations we found in this study may be explained by the fact that chronic inflammation, especially in the pancreas, raises the degree of tumor fibrosis by upregulating epithelial–mesenchymal transition (EMT) (24, 25). The fibrous tissue obstructs and delays the entry of contrast agent, resulting in less enhancement. Hattori et al. also found that the degree of fibrosis within PDAC tumors was inversely correlated with tumor enhancement on contrast-enhanced CT scans, and the correlation was more significant in the arterial and venous phases (26). However, we found that the AUCs for tumor enhancement in the arterial and venous phases were only 0.542 and 0.570, indicating that although tumor enhancement was correlated with inflammatory infiltration, it could not be used to predict inflammatory infiltration. This may be due to the limitation of naked eye observation and evaluation of CT images. In contrast, the rapid development of texture analysis in recent years can overcome limitations and extract a massive amount of quantitative information directly from image data, leading to more desirable predictive performance (8, 9). Moreover, in a retrospective study, Toft et al. demonstrated that MRI has higher accuracy, sensitivity, and specificity than CT in the diagnosis of pancreatic cancer (27), which is due to the higher soft tissue resolution of MRI. This suggests that the correlations and predictive performance of MRI scanning with indicators related to the tumor inflammatory microenvironment can be further explored in future studies.

Since the predictive value of conventional CT imaging is limited, we continued to investigate the value of contrast-enhanced CT images in assessing inflammatory infiltration within PDAC tumors using texture analysis; the Variance Threshold and SelectKBest algorithms were used to select the best texture features for inflammatory infiltration at each of contrast-enhanced CT scan phase, and logistic regression equations were established using the screened features. Comparing the ROC curves of the regression equations obtained for each phase, we found that the equation based on the optimal features from the arterial phase had the highest AUC among the phases (0.982), as well as the highest sensitivity (100%) and specificity (89.5%), demonstrating a promising predictive value; the delayed phase had the second highest AUC of 0.849, showing moderate predictive performance; and the venous phase had the lowest AUC of 0.643, showing poor predictive performance. Therefore, the arterial phase may be the optimal phase for performing texture analysis in our study, which may be related to the fact that the arterial phase magnifies the difference between tumor and normal tissue (28–30). Based on this, the inflammatory infiltration degree of PDAC can be assessed more accurately with data from this phase.

Our study had certain limitations. First, the sample size was relatively small, which may lead to bias in the results; Second, the ROIs were outlined only on images containing the maximum cross- sections of the tumor, and therefore we were unable to perform 3D whole-tumor analysis. Finally, we did not correlate imaging features with clinical symptoms, biochemical indicators, and prognostic indices.

In summary, conventional CT imaging analysis has some limitations in predicting inflammatory infiltration within PDAC tumors, while texture analysis based on contrast-enhanced CT images has better predictive performance for inflammatory infiltration, especially in the arterial phase.

## Data availability statement

The raw data supporting the conclusions of this article will be made available by the authors, without undue reservation.

## Ethics statement

The studies involving human participants were reviewed and approved by Ethics Committee on Scientific Research of Shandong University Qilu Hospital. The ethics committee waived the requirement of written informed consent for participation.

## Author contributions

DY contributed to conception and design of the study. FW and HG organized the database. JX performed the statistical analysis. HG wrote the first draft of the manuscript. FW wrote sections of the manuscript. All authors contributed to manuscript revision, read, and approved the submitted version.
